# Advance in Medicine as a Science

**Published:** 1872-08

**Authors:** 


### EDITORIAL AND MISCELLANEOUS.

### ADVANCE IN MEDICINE AS A SCIENCE.

The evidences of progress in medical science, to the intelligent observer, are readily found in all its departments, but are most conspicuously exhibited in connection with cardiac diseases. We find in the July number, for 1872, of the *London Lancet* the following editorial, which is in such striking accord with our own views and observations, and is so forcible and plain an exposition of the present status of heart diseases, that we transfer it to our pages, being assured that nothing could occupy the space more to the pleasure, profit or honor of the profession. With the present knowledge and skill in the treatment of cardiac diseases, there is no longer any justification for the wholesale condemnation to speedy and sudden death, to which patients affected with diseases of the heart were formerly subjected. Indeed, our knowledge in regard to the pathology and therapeutics of this subject forms one of the most cheerful and interesting pages in the history of the progress of medical science. We can not regret that by these results the number of skillful prognosticators, in regard to the certain and speedy fatality of diseases of this class, has been greatly diminished in proportion to the progress in accuracy of the knowledge acquired by the diagnostician, the pathologist, and therapeutist.

Diseases of the Heart.—The subject of diseases of the heart is one of great interest, both practical and pathological. It is, comparatively speaking, a new subject. The older pathologists gave the heart itself very little consideration. There was only one criterion to them of its action—namely, the pulsation of the radial artery. Almost their whole attention was given to the blood which the heart pumped through the body; the condition of the pump itself was almost ignored. The diseases of the lungs were not so much overlooked. They rendered themselves more palpable by various objective symptoms, although the differentiation of the various diseases of these organs was only accomplished by the discoveries of Laennec, which have also enabled physicians to test the condition of the heart by various physical methods. It is very interesting to notice how little is made of the heart by older authors. Heberden discusses palpitation, and obviously regarded it as occasionally having relations with grave constitutional states, such as those produced by too much care and business, asthma, gout, and general failure of the powers of life known by the name of a broken constitution. He gives, as an appendix to his Commentaries, an interesting paper entitled "Remarks on the Pulse," in which he expresses himself dissatisfied with the minute distinctions of the several pulses made by physicians, saying that they do not exist save in the imagination; and suggests that they should attend only to such circumstances concerning the pulse as can not be misunderstood, and he specifies the frequency of the pulse as a circumstance that could be accurately noted, and should be chiefly attended to. Heberden clearly sighed for accuracy, and thought this quality could attach only to observations of the number of pulsations. Cullen scarcely seems to have thought of the heart as an organ capable of beng affected with structural disease.

The detection and differentiation of the diseases of the heart; the relation of the most common of these to acute rheumatism; the study of morbid changes in the muscular structure of the heart; and the perception of the high importance of attending, even in cases of valvular disease, largely if not mainly to the condition of the muscular tissue; these are a few of the most characteristic and creditable points in the medical work of the present century. It is curious that Heberden, who, with great sagacity, as we have shown, specified the serious constitutional condition with which palpitation was frequently associated, contrived to leave out rheumatism. The honor of the discovery of the connection of acute rheumatism with inflammations of the heart is, we believe, due to Dr. David Pitcairn, who first noticed the fact in 1788; but it was not until Sir D. Dundas' account of the disease called "rheumatism of the heart," in 1809, that this great pathological relation was made distinct, which is now so well understood by every tyro in medicine. The detection and differentiation of heart disease could not have advanced as it has done apart from the introduction of the stethoscope by Laennec. But it is gratifying to know that English, Irish and Scotch physicians have greatly distinguished themselves by their labors in this branch of pathology, especially that the researches of Pitcairn, Dundas, Bright, Latham and Hope have been brilliantly continued by Stokes, Corrigan, Graves, Quain, George Johnson, Richardson, Fothergill, and a host of others.

It is not our intention to pursue this line of thought further. But if anybody doubts the progress of medical science, let him compare the knowledge possessed by physicians in the beginning of this century, of the morbid conditions of the heart, with the knowledge possessed by physicians now, and he will admit that the advance is enormous. An ungenial critic may say, *cui bono?* To what end is all the elucidation of the different lesions of the different tissues, if it does not lead to a more enlightened and efficient treatment? But this is just what it does lead to, and there can be no doubt that the medical treatmeht of diseases of the heart is much improved. It is especially improved in two or three respects. In the first place, much more attention is devoted to other local disorders and to the constitutional states with which heart disease is associated. By a proper medical regard to these, the suffering heart is often either completely relieved or greatly improved. Secondly, the physician of the present day directs his treatment to improving the vital tissue—the muscle—of the heart, and does not aim at impracticable alterations of the condition of the valves. He knows that much may be done to nourish, to rest, to save the muscular tissue, and his treatment has these objects chiefly in view. Moreover, the use and action of cardiac medicines are much more accurately defined than they were. Surely, too, we may hope that, despite the sceptics, a better treatment of rheumatic fever does tend to save the heart—to save its muscular tissue, if not its valves. There is a third grand advantage in modern medicine: it can take a more hopeful view, and give a more hopeful prognosis. We do not think it necessary to imitate that physician of whom Dr. Stokes has somewhere spoken, who, on hearing a bruit in a patient's heart, who up to that time had been working very well notwithstanding his bruit, said—"Sir, I hear your death-knell;" and so felled the patient with prognosis. We see too much of the recovery of heart-power by rest, food, and proper medicines; we know too many pulses that are beating happily now, that we knew to intermit or to be irregular years ago; we know, on such authority as that of Dr. Stokes, that the patient with heart disease does not, in the great proportion of cases, die suddenly, but gradually, and with a failure that may, by care on his part, and a due regard to medical hints and helps, be greatly retarded and averted. On such grounds as these, heart disease has come to be a creditable and hopeful chapter in medical science.

